# The Effect of Three Eye Care Methods on the Severity of Lagophthalmos in Intensive Care Patients: A Randomized Controlled Clinical Trial

**DOI:** 10.1155/2021/6348987

**Published:** 2021-09-15

**Authors:** Tahere Nikseresht, Mansour Rezaei, Alireza Khatony

**Affiliations:** ^1^Student Research Committee, Kermanshah University of Medical Sciences, Kermanshah, Iran; ^2^Social Development and Health Promotion Research Center, Health Institute, Kermanshah University of Medical Sciences, Kermanshah, Iran; ^3^Infectious Diseases Research Center, Kermanshah University of Medical Sciences, Kermanshah, Iran

## Abstract

**Background:**

Patients admitted to intensive care units are exposed to a variety of eye injuries such as lagophthalmos, which can lead to blindness. There is conflicting evidence regarding the effectiveness of different eye protection methods, and evaluations are ongoing. Therefore, this study was performed to compare the effect of “polyethylene cover,” “polyethylene cover plus artificial tear drops,” and “polyethylene cover plus Lubratex eye ointment” on the severity of lagophthalmos.

**Methods:**

A total of 156 patients connected to ventilators were included in this clinical trial using the convenience sampling method. They were randomly divided into three groups: “polyethylene cover,” polyethylene cover plus artificial tear drops,” and “polyethylene cover plus Lubratex eye ointment.” In each group, one eye was regarded as control and the other eye as intervention. The control eye received routine interventions, including washing with normal saline. The eyes were examined daily by an ophthalmologist for 5 days for the occurrence of lagophthalmos.

**Results:**

There was no statistically significant difference in the severity of lagophthalmos among the three groups “polyethylene cover,” “polyethylene cover plus artificial tear drop,” and “polyethylene cover plus Lubratex eye ointment.” However, clinically the severity of lagophthalmos was lower in the “polyethylene cover plus artificial tear drops” group than in the other two groups.

**Conclusion:**

The results showed that the combination of polyethylene cover and artificial tears drops can be clinically effective in reducing the severity of lagophthalmos. Therefore, the use of this method is recommended for patients admitted to the intensive care unit. Similar studies are recommended.

## 1. Introduction

Patients admitted to intensive care units are exposed to a variety of eye injuries, including lagophthalmos, for a variety of reasons such as the use of sedatives and muscle relaxants, metabolic causes, and mechanical ventilation [1–4]. Lagophthalmos means the inability of the eyelids to close completely or incompletely due to the paralysis of muscles around the eyes caused by the use of sedatives and neuromuscular relaxants [5]. Lagophthalmos is the main cause of ocular surface disorder (OSD) in intensive care unit (ICU) patients [1, 3, 6]. Studies have shown that 60% of the ICU patients who have tracheal tubes and suffer from lagophthalmos are at risk of serious eye injuries [7]. Evidence suggests that patients with lagophthalmos also have some degrees of chemosis [8]. Kam and Mercieca reported the incidence of chemosis and lagophthalmos in the ICU patients to be 75% and 80–90%, respectively [3, 5]. Therefore, it is important to check the position of eyelids and their closure and openness in critically ill ICU patients [1]. Various studies have been performed on the effect of eye care on the incidence of lagophthalmos [2–4, 6]. There are various methods to protect the eyes in ICU patients, including the use of eye drops and ointments, the creation of a moisture chamber with normal saline-soaked gauze, the creation of a closed chamber using polyethylene or swimming goggles, and the adhesion of eyelids [9]. Different studies have compared the efficacy of these care methods in preventing superficial eye injuries and have shown contradictory results [10–13]. In the study of Babamohamadi et al. [10], the effects of vitamin A ointment and moisture chamber in the prevention of ocular surface disorders were compared, the results of which showed both methods were ineffective [10]. Ezra et al. compared the effects of ocular lubricants and polyacrylamide hydrogel dressings on the prevention of exposure keratopathy in ICU patients, both of which were equally effective [11]. In a study by Ghanei et al. [14], the effects of the three methods moist gauze, eyelid adhesion, and eye ointment on the prevention of OSD were compared, and the results showed all three methods had the same effect [14]. In a case study, the effect of kinesiology tape on lagophthalmos in a 26-year-old woman with grade 3 burns was investigated, and the results showed that this method was effective [15]. In another study, Gervasio et al. [16] compared the effects of “prosthetic replacement of the ocular surface ecosystem” and “standard care” methods on the severity of lagophthalmos after surgery, and the results showed that the standard care method was more effective [16]. In this regard, the results of a meta-analysis comparing the efficacy of the two methods moisture chamber and ocular lubricant in the prevention of OSD showed the effectiveness of the moisture chamber method [17]. Koroloff et al. [18] investigated the effect of hypromellose and Lacri-Lube combination versus polyethylene/cling wrap on the prevention of OSD. Their results showed that both methods did not affect the severity of lagophthalmos [18]. Cortes et al. [19] investigated the effect of the two methods polyethylene cover and methylcellulose lubricant drops on the prevention of OSD. They showed that the lubricant drops were more effective [19].

Given the conflicting evidence regarding the effectiveness of various eye protection methods in ICU patients, studies and evaluations are still ongoing in this field [17]. Considering this issue and given that lagophthalmos is a very common ocular complication in ICU patients that can lead to blindness [4], the researchers in this study decided to study the effect of “polyethylene cover,” “polyethylene cover with artificial tear drops,” and “polyethylene cover with Lubratex eye ointment” on the severity of lagophthalmos in ICU patients.

## 2. Materials and Methods

### 2.1. Study Design

This study was designed as a prospective, single-blind, clinical trial.

### 2.2. Study Hypothesis

“Polyethylene cover,” “polyethylene cover and artificial tear drops,” and “polyethylene cover and Lubratex eye ointment” have different effects on the severity of lagophthalmos.

### 2.3. Sample and Sampling Method

The study population consisted of all intubated patients with loss of consciousness in the ICUs of teaching hospitals in Ilam-Iran. The sample size was estimated to be 156 using the results of the studies of Cortese et al. [19] and Koroloff et al. [18], with 95% confidence level and 80% power. Patients who met inclusion criteria were selected and randomly divided into three groups—“polyethylene cover,” “polyethylene cover and artificial tear drops,” and “polyethylene cover and Lubratex eye ointment” (52 in each group)—using the random number table. In each sample, one eye was considered the intervention eye and the other eye was considered the control eye. The choice of each eye as intervention and control was random and done through the random number table.

The inclusion criteria were age ≥ 18 years, Glasgow Coma Scale (GCS) score ≤8, connection to a mechanical ventilator using a tracheal tube, absence of superficial eye diseases (based on negative results of Schirmer and fluorescein tests), no use of ophthalmic drugs, no trauma to the face, hospitalization in the ICU for more than 24 hours, and incomplete eyelid closure. Exclusion criteria were increased level of consciousness, extubation, reversal of the blink reflex, transfer to another ward, and death.

### 2.4. Measurement Instruments

Data collection tools included a personal information form, lagophthalmos intensity checklist [13], HSL150 hand-held slit lamp (made in Germany), graded sterile strips (SM Tube, Japan), sterile fluorescein stain strips (Elham Teb, Iran), polyethylene cover (Pars Atlas, Iran), artificial tear drops (Tearlose), and eye ointment (Lubratex; Sina Darou, Iran).

The demographic information form contained 14 questions, 13 of which were on demographic characteristics, diagnosis, Glasgow Coma Scale, length of hospital stay, use or absence of anesthetic-sedative drugs, amount and duration of use, peak inspiratory pressure (PIP), and positive end-expiratory pressure (PEEP). Question 14 was about the “grade of eyelid position or severity of lagophthalmos” in each of the control and intervention eyes, respectively.

To determine the intensity of lagophthalmos, the grading table for eyelid position was used, which graded the intensity of lagophthalmos from zero to 5: grade “zero” for “complete closure of the eyelids,” grade “one” for only conjunctival exposure, grade “two” for “ exposure of lower 1/4th of cornea,” grade “three” for “exposure of 1/2nd of cornea,” grade “four” for “exposure of 3/4th of cornea,” and grade “five” for full corneal exposure [13]. Schirmer and fluorescein tests were used to rule out the dry eye and corneal abrasion, respectively.

### 2.5. Interventions

This study was part of a large study. First, the necessary permissions were obtained from the Kermanshah University of Medical Sciences. Then, the researcher selected the patients from among the eligible patients admitted to the ICUs of Imam Khomeini and Shahid Mostafa Khomeini teaching hospitals in Ilam-West of Iran. These hospitals are the main centers for internal medicine and trauma, each with two ICUs with 24 active beds.

The absence of dry eye and corneal abrasion in eligible patients were evaluated and confirmed by an ophthalmologist. Schirmer and fluorescein staining tests were used to evaluate dry eye and corneal abrasion, respectively. In the Schirmer test, the tip of the tape was placed at the edge of the lower eyelid. The wetness of the tape up to 5 mm or more indicated the lack of dryness. After ruling out the dryness, to perform fluorescein staining, a sterile paper strip containing the orange fluorescein was gently stretched to the inner edge of the lower eyelid. Then, the eyelids were opened and closed several times by hands to spread the dye on the eye surface. The ophthalmologist then examined the surface of the cornea using the blue light of a slit lamp. Having confirmed the absence of dry eye and corneal abrasion, the patients were randomly divided into three groups: “polyethylene cover,” “polyethylene cover and artificial tear drops,” and “polyethylene cover and Lubratex eye ointment” (52 patients in each group).

In the intervention eye of the “polyethylene cover” group, the cover was stretched by the researcher from the top of the eyebrow to the cheekbone ridge, and the surrounding area was tightened with an antiallergic adhesive. In the “polyethylene cover and artificial tear drops” group, a drop of artificial tear was inserted into the lower eyelid of the intervention eye by the researcher every eight hours, and then the polyethylene cover was stretched from the top of the eyebrow to the ridge of the cheekbone. Then, its surrounding was tightened with an antiallergy adhesive. In the “polyethylene cover and Lubratex eye ointment” group, a layer of ointment with a diameter of one centimeter was applied into the lower eyelid every eight hours, and then the polyethylene cover was stretched from the eyebrow to the ridge of the cheekbone and tightened with an antiallergic adhesive. In all three groups “polyethylene cover,” “polyethylene cover and artificial tear drops,” and “polyethylene cover and Lubratex eye ointment,” the polyethylene cover was replaced daily by the researcher. However, in case of rupture, damage, contamination, or removal of the polyethylene cover from the skin surface, it was immediately replaced by the researcher.

**Figure 1:**
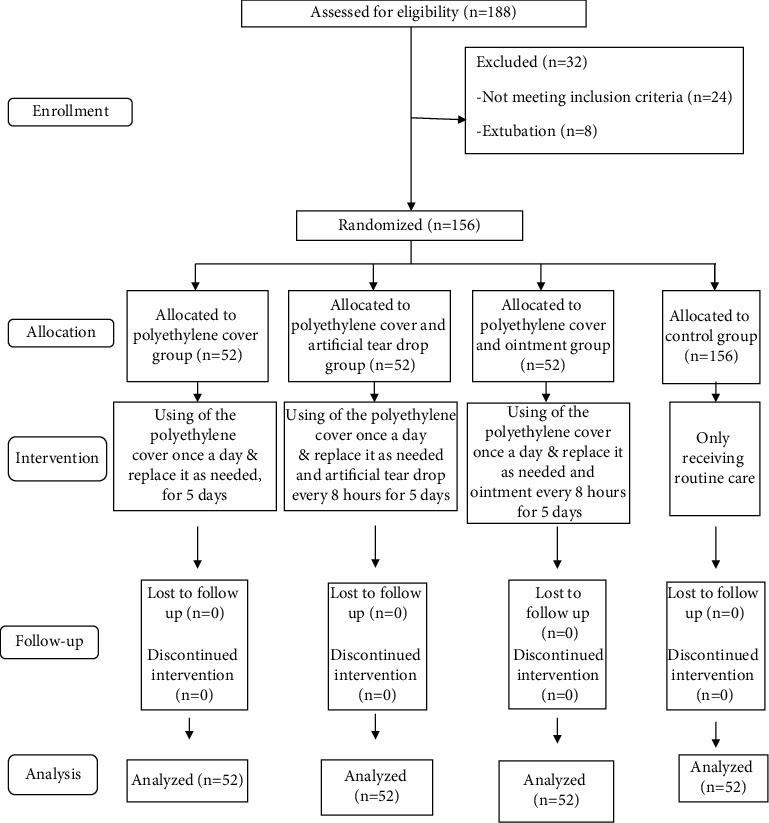
Flow diagram of the study.

The control eyes in all three groups “polyethylene cover,“ “polyethylene cover and artificial tear drops,” and “polyethylene cover and Lubratex eye ointment” received routine care. These treatments included rinsing the inside of the eyes, eyelids, and surrounding area with distilled water and then drying with sterile gauze, which was performed by the researcher every eight hours. The intervention took five days, during which the ophthalmologist used a slit lamp to examine the patients' “intervention” and “control” eyes in all three groups daily in terms of the grade of lagophthalmos and recorded the results of the examination in the relevant forms (Figure 1).

### 2.6. Data Analysis

Data were analyzed using SPSS V.18 software. First, the normality of the data was measured using the Kolmogorov–Smirnov test. Because all study variables except age had an abnormal distribution, nonparametric tests were used to analyze the data. One-way analysis of variance (ANOVA) was used to compare the mean age of the three study groups. The Friedman test was used to evaluate the severity of lagophthalmos in each of the control and intervention eyes during 5 days. The Wilcoxon test was used to compare the grade of lagophthalmos between the intervention and control eyes in each group. The Kruskal–Wallis test was used to compare the severity of lagophthalmos between the intervention eyes in the study groups. The significance level was set at <0.05.

### 2.7. Ethical Considerations

This study was conducted in accordance with the Helsinki Declaration. The study was approved by the Ethics Committee of the Kermanshah University of Medical Sciences (code: 1395.177.KUMS.REC) and was registered in the Iranian Registry of Clinical Trials (code: IRCT201506294736N8). Since the patients under study were not conscious and were connected to a ventilator, written informed consent was obtained from their first-degree relatives (parents or legal guardians) and they were assured that their personal information about the patients' data would be kept confidential.

## 3. Results

Out of 156 samples, 113 (73%) were male. The mean age of the patients was 52.8 ± 21.1 years. The mean length of hospitalization in the ICU was 9.0 ± 2.0 days. There was no statistically significant difference among the three groups “polyethylene cover,” “polyethylene cover and artificial tear drops,” and “polyethylene cover and Lubratex eye ointment” in terms of demographic characteristics and intervening variables, including the type of disease, length of hospitalization, GCS, use or nonuse of anesthetic-sedative drugs, amount and duration of anesthetic-sedative drugs, PIP, and PEEP (Table 1).

In the “polyethylene cover” group, the severity of lagophthalmos in the intervention eye increased from the first to the fifth day of the study. The severity of lagophthalmos was significantly different at the end of the fifth day compared with the first day (*P* < 0.001). In the control eye, lagophthalmos increased from day 1 to day 4 but decreased on day 5 compared with day 4. In this group, the severity of lagophthalmos was significantly different at the end of the fifth day compared with the first day (*P* < 0.001) (Table 2).

Regarding the severity of lagophthalmos, there was a statistically significant difference between the intervention and control eyes only on the first day from among the 5 days of the study (*P*=0.03), which indicated that the use of polyethylene cover in the intervention eye had no significant effect on lagophthalmos compared with the routine care in the control eye (Table 3).

In the “polyethylene cover and artificial tear drops” group, the severity of lagophthalmos in the intervention eye increased from the first to the fourth day, but it was less intense on the fifth day compared with the fourth day. Yet, there was no statistically significant difference between the fifth day and the first day in terms of the progression of lagophthalmos severity in the intervention eye. In the control group, lagophthalmos progressed to higher grades from the first day to the fourth day but decreased on the fifth day compared with the fourth day. In this group, there was no significant difference in the severity of lagophthalmos at the end of the fifth day compared with the first day. The results indicated no statistically significant difference in the severity of lagophthalmos between the intervention and control eyes from the first to fifth days of the study. This result showed that the use of polyethylene cover and artificial tear drops in the intervention eye had no effect on the severity of lagophthalmos compared with the routine care in the control eye (Table 3).

In the “polyethylene cover and Lubratex ocular ointment” group, the severity of lagophthalmos in the intervention eye increased from the first to the fourth day, but it was less severe on the fifth day compared with the fourth day. In this group, there was a statistically significant difference in the severity of lagophthalmos between the fifth day and the first day (*P* < 0.001). In the control eye, the severity of lagophthalmos increased from the first to the fourth day but decreased on the fifth day compared with the fourth day. In this group, there was also a statistically significant difference in the severity of lagophthalmos between the fifth and the first days (*P* < 0.001). There was no statistically significant difference in the severity of lagophthalmos between the intervention and control eyes during the study period, but clinically, the severity of lagophthalmos was lower than in the intervention eye than in the control eye at the end of the fifth day. This result indicated that the use of “polyethylene cover plus Lubratex eye ointment” in the intervention eye could reduce the severity of lagophthalmos compared with the routine care in the control eye (Table 2).

During the study days, there was no statistically significant difference between the intervention and control eyes in terms of the severity of lagophthalmos, but clinically, the severity of lagophthalmos was lower in the intervention eye than the in control eye at the end of the fifth day of the study (Table 3). The results showed no statistically significant difference in the severity of lagophthalmos among the three groups “polyethylene cover,” “polyethylene cover and artificial tear drop,” and “polyethylene cover and Lubratex eye ointment.” Clinically, the severity of lagophthalmos was lower in the “polyethylene cover plus artificial tear drops” group than in the other two groups (Table 4).

## 4. Discussion

This study was aimed to compare the efficacy of three eye care methods, including “polyethylene cover,“ “polyethylene cover with artificial tear drops,” and “polyethylene cover with Lubratex eye ointment,” on the severity of lagophthalmos in ICU patients. The results showed no statistically significant difference among the three groups “polyethylene cover,” “polyethylene cover and artificial tear drops,” and “polyethylene cover and Lubratex eye ointment” in terms of the severity of lagophthalmos. Few studies have investigated the effect of different eye care methods on the severity of lagophthalmos, which have shown different results. In this regard, a study compared the effectiveness of the use of vitamin A eye ointment and moisture chamber in preventing OSD in 38 ICU patients over 5 days. The results showed that both methods were ineffective in the prevention of lagophthalmos [10]. In this study, the eye care methods included open chamber (Lacri-Lube ointment) and closed chamber (polyethylene cover). But in our study, all three eye care methods were of closed chamber type (polyethylene cover) or of moisture chamber type (a combination of polyethylene cover and artificial tear drops or eye ointment). Although the results of both studies are similar, our study showed that the combination of polyethylene cover and artificial tears drops can be clinically effective in reducing the severity of lagophthalmos. In other words, despite the lack of a statistically significant difference among the three care methods, clinically, the severity of lagophthalmos was lower in the “polyethylene cover plus artificial tear drops” group than in the other two groups. Based on this result, the use of this method can be recommended for patients at risk of lagophthalmos.

In a clinical trial, the effect of prosthetic replacement of the ocular surface ecosystem was compared with that of standard care methods on the severity of lagophthalmos and keratopathy in 45 patients after skull surgery. The results showed that the use of standard methods such as artificial tear drops or eyelid adhesion was more effective in the treatment of lagophthalmos [16]. One of the standard care methods used in this study was polyethylene cover, which is one of the care methods affecting lagophthalmos. In our study, polyethylene cover was used in all three methods, and there was no statistically significant difference in the severity of lagophthalmos among the three groups “polyethylene cover,” “polyethylene cover plus artificial tear drop,” and “polyethylene cover plus Lubratex eye ointment.”

A clinical trial compared the efficacy of adhesive, antibiotic ointment, artificial tears, and ocular lubricant ointment in preventing ocular injuries in 184 patients under general anesthesia. The results showed that the amount of ocular injuries was higher in the adhesive group than in the other three groups [12]. In this study, the four care methods were of open chamber type, but in our study, all three methods were of closed or moisture chamber type. Another study compared the effects of ocular lubricant ointment and passive closure of the eyelid on corneal abrasion in 50 ICU patients. The results showed that the severity of lagophthalmos was the same in both groups [4]. Furthermore, a study compared the effects of “hypromellose drop and Lacri-Lube” and “polyethylene coating” on corneal epithelial damage in 110 ICU patients. The results revealed 5% in the polyethylene group and 7% in the “hypromellose and Lacri-Lube” group had lagophthalmos, which indicated both methods had the same effect on the severity of lagophthalmos [18]. In this study, two methods of closed chamber (polyethylene cover) and moisture chamber (eye lubricant) were compared, but our study included a closed chamber method (polyethylene cover) and two moisture chamber methods (polyethylene cover plus artificial tear drops or eye ointment). In our study, similar to this study, there was no statistically significant difference among the eye care methods in reducing the severity of lagophthalmos. However, the results showed that the combination of “polyethylene cover and artificial tear drops” can be clinically effective in reducing the severity of lagophthalmos. The combined use of artificial tear drops and polyethylene cover, by creating a closed chamber on the eye, prevents the evaporation of tear fluid and maintains the moisture of the eye surface. This method of care also reduces the severity of the lagophthalmos by relaxing the muscles that support the eyelid [20, 21].

## 5. Limitations

Two of the major limitations of this study were ignoring the age limit and employing a short study period due to limited resources and facilities. Moreover, most of our patients were brain injury patients who needed constant control of pupillary light reflex in every shift, which interfered with the polyethylene cover. In such cases, the polyethylene cover was removed and reinserted or replaced if necessary to examine the pupils.

## 6. Conclusion

The results of this study showed that there was no statistically significant difference among the three study groups in terms of the severity of lagophthalmos in ICU patients. However, “polyethylene cover and artificial tear drops” can be clinically effective in reducing the severity of lagophthalmos in this group of patients. Considering the results of the few studies conducted in this regard, further research is needed to compare the effect of care methods used for the prevention of ocular complications such as lagophthalmos in ICU patients. Similar studies are also recommended to consider a longer study period and take into account the age limit.

## Figures and Tables

**Table 1:** Demographic characteristics of patients.

Variables	Polyethylene cover (*n* = 52)	Polyethylene cover with artificial tear drop (*n* = 52)	Polyethylene cover with eye ointment (*n* = 52)	*P-value*
Age (mean ± SD)	53.0 ± 20.5	51.3 ± 19.7	4.20 ± 1.54	NS^†^
Sex (male:female)	33.6 : 32.6	32.7 : 34.9	33.6 : 32.6	NS
Marital status (single:married)	32.5 : 33.6	45.0 : 29.0	22.5 : 37.0	NS
ICU^‡^ length of stay (mean ± SD)	10.0 ± 3.0	9.0 ± 2.0	9.0 ± 20.0	NS
GCS^§^ (mean ± SD)	5.0 ± 1.0	6.0 ± 1.0	6.0 ± 1.0	NS
Days of sedation (mean ± SD)	0.0 ± 1.0	1.0 ± .1.0	1.0 ± 1.0	NS
Use of sedation (% of patients)	27.0	39.0	34.0	NS
PIP^ǁ^ (mean ± SD)	21.0 ± 4.8	21.0 ± 4.0	20.0 ± 2.0	NS
PEEP^‡‡^ (mean ± SD)	5.0 ± 1.0	0.0 ± 5.0	5.0 ± 0.0	NS
Diagnosis (trauma:neurosurgical:medical)	27 : 27 : 40	41.9 : 36.4 : 25	30.6 : 36.4 : 34.7	NS

^†^ NS: nonsignificant; ^‡^ ICU: intensive care unit;^§^ GCS: Glasgow Coma Scale; ^ǁ^PIP: peak inspiratory pressure; ^‡‡^PEEP: positive end-expiratory pressure.

**Table 2:** Comparison of lagophthalmos severity*∗* in each study group from the first to the fifth day of the study.

Study days	Polyethylene cover				Polyethylene cover & artificial tear drop				Polyethylene cover & eye ointment			
	Intervention group	*P*-value	Control group	*P*-value	Intervention group	*P*-value	Control group	*P*-value	Intervention group	*P*-value	Control group	*P*-value

First	2.4	<0.001^**†**^	2.5	<0.001^**†**^	2.8	0.286	2.8	.074	2.7	<0.001^**†**^	2.6	<0.001^**†**^
Second	2.8		2.6		2.9		2.9		2.7		2.6	
Third	3.1		3.1		2.9		2.9		3.1		3.2	
Fourth	3.3		3.5		3.2		3.2		3.4		3.4	
Fifth	3.4		3.4		3.1		3.2		3.2		3.2	

Note: † There was a significant difference between the first and fifth days of the study in terms of the severity of lagophthalmos. ^*∗*^To determine the severity of lagophthalmos, the grading table for eyelid position was used, which graded the intensity of lagophthalmos from zero to five: grade “zero” for “complete closure of the eyelids,” grade “one” for only conjunctival exposure, grade “two” for “ exposure of lower 1/4th of cornea,” grade “three” for “exposure of 1/2nd of cornea, ” grade “four” for “exposure of 3/4th of cornea,” and grade “five” for full corneal exposure.

**Table 3:** Comparison of lagophthalmos severity*∗* between intervention and control eyes from the first to the fifth day of the study.

Study days	Polyethylene cover		*P*-value	Polyethylene cover & eye drop		*P*-value	Polyethylene cover & ointment		*P*-value
	Intervention (*n* = 52)	Control (*n* = 52)		Intervention (*n* = 52)	Control (*n* = 52)		Intervention (*n* = 52)	Control (*n* = 52)	

First	6.1	5.5	0.033	3.0	3.0	0.655	3.7	2.5	1
Second	8.3	8.7	0.936	5.0	4.0	0.763	5.0	4.0	0.773
Third	7.6	10.7	0.151	6.4	5.5	0.593	3.9	4.2	0.340
Fourth	7.8	6.6	0.092	6.4	6.7	0.655	5.4	6.2	0.331
Fifth	6.0	6.0	0.132	7.1	6.8	0.396	3.2	4.0	0.589

^*∗*^To determine the severity of lagophthalmos, the grading table for eyelid position was used, which graded the intensity of lagophthalmos from zero to five: grade “zero” for “complete closure of the eyelids,” grade “one” for only conjunctival exposure, grade “two” for “ exposure of lower 1/4th of cornea,” grade “three” for “exposure of 1/2nd of cornea,” grade “four” for “exposure of 3/4th of cornea,” and grade “five” for full corneal exposure.

**Table 4:** Comparison of the severity of lagophthalmos in the eyes of the intervention group at the end of the fifth day.

Lagophthalmos severity	Study groups			*P*-value
	Polyethylene cover (*n* = 52)	Polyethylene cover with eye drop (*n* = 52)	Polyethylene cover with ointment (*n* = 52)	
	79.43	76.76	79.31	0.935

## Data Availability

The identified data sets analyzed during the current study are available from the corresponding author on reasonable request.

## Abbreviations

OSD: Ocular surface disorder
ICU: Intensive care unit
PIP: Peak inspiratory pressure
PEEP: Positive end-expiratory pressure.

## References

[1] Alansari M. A., Hijazi M. H., Maghrabi K. A. (2015). Making a difference in eye care of the critically ill patients. *Journal of Intensive Care Medicine*.

[2] Bendavid I., Avisar I., Serov Volach I. (2017). Prevention of exposure keratopathy in critically ill patients: a single-center, randomized, pilot trial comparing ocular lubrication with bandage contact lenses and punctal plugs. *Critical Care Medicine*.

[3] Kam K. R., Haldar S., Papamichael E., Pearce K. C., Hayes M., Joshi N. (2013). Eye care in the critically ill: a national survey and protocol. *Journal of the Intensive Care Society*.

[4] Lenart S., Garrity J. (2000). Eye care for patients receiving neuromuscular blocking agents or propofol during mechanical ventilation. *American Journal of Critical Care*.

[5] Gregory D. (2003). *Eye Care for Patients in Critical Care Units*.

[6] Demirel S., Cumurcu T., Fırat P., Aydogan M. S., Doğanay S. (2014). Effective management of exposure keratopathy developed in intensive care units: the impact of an evidence based eye care education programme. *Intensive and Critical Care Nursing*.

[7] McHugh J., Alexander P., Kalhoro A., Ionides A. (2008). Screening for ocular surface disease in the intensive care unit. *Eye*.

[8] Mercieca F., Suresh P., Morton A., Tullo A. (1999). Ocular surface disease in intensive care unit patients. *Eye*.

[9] Marshall A. P., Elliott R., Rolls K., Schacht S., Boyle M. (2008). Eyecare in the critically ill: clinical practice guideline. *Australian Critical Care*.

[10] Babamohamadi H., Nobahar M., Razi J., Ghorbani R. (2018). Comparing vitamin A and moist chamber in preventing ocular surface disorders. *Clinical Nursing Research*.

[11] Ezra D. G., Chan M. P. Y., Solebo L. (2009). Randomised trial comparing ocular lubricants and polyacrylamide hydrogel dressings in the prevention of exposure keratopathy in the critically ill. *Intensive Care Medicine*.

[12] Kocatürk Ö, Kocatürk T., Kaan N., Dayanır V. (2012). The comparison of four different methods of perioperative eye protection under general anesthesia in prone position. *Yüzüstü Pozisyonda Genel Anestezi Altında Peroperatif Göz Korumada Dört Ayrı Yöntemin Karşılaştırılması*.

[13] Sivasankar S., Jasper S., Simon S., Jacob P., John G., Raju R. (2006). Eye care in ICU. *Indian Journal of Critical Care Medicine*.

[14] Ghanei M., Matine S., Radmehr M., Pakdel M., Kalani N. (2016). Comparison of three methods of wet gauze, adhesive tape and eye ointment to prevent corneal ulceration in patients undergoing general anesthesia. *Journal of Fundamental and Applied Sciences*.

[15] Pauley J., Schnake E., Smith T., Lambert Wagner A., Wiktor A. J. (2018). 496 treatment of lagophthalmos using kinesiology tape in burn patients: a case study. *Journal of Burn Care and Research*.

[16] Gervasio K. A., Godfrey K. J., Marlow E. D., Lee M. N., Lelli G. J. (2019). Prosthetic REPLACEMENT of the ocular surface ecosystem (PROSE) versus standard of care for postsurgical lagophthalmos and exposure keratopathy: trends in visual outcomes. *Ophthalmic Plastic and Reconstructive Surgery*.

[17] Zhou Y., Liu J., Cui Y., Zhu H., Lu Z. (2014). Moisture chamber versus lubrication for corneal protection in critically ill patients: a meta-analysis. *Cornea*.

[18] Koroloff N., Boots R., Lipman J., Thomas P., Rickard C., Coyer F. (2004). A randomised controlled study of the efficacy of hypromellose and Lacri-Lube combination versus polyethylene/Cling wrap to prevent corneal epithelial breakdown in the semiconscious intensive care patient. *Intensive Care Medicine*.

[19] Cortese D., Capp L., McKinley S. (1995). Moisture chamber versus lubrication for the prevention of corneal epithelial breakdown. *American Journal of Critical Care*.

[20] Shan H., Min D. (2010). Prevention of exposure keratopathy in intensive care unit. *International Journal of Ophthalmology*.

[21] So H. M., Lee C. C. H., Leung A. K. H., Lim J. M. J. A., Chan C. S. C., Yan W. W. (2008). Comparing the effectiveness of polyethylene covers (Gladwrap™) with lanolin (Duratears®) eye ointment to prevent corneal abrasions in critically ill patients: a randomized controlled study. *International Journal of Nursing Studies*.

